# Supplementary material for: Viruses in unexplained encephalitis cases in American black bears (Ursus americanus)
Source: PLoS One. 2020 Dec 17;15(12):e0244056. doi: 10.1371/journal.pone.0244056 (PMC7745964; doi:10.1371/journal.pone.0244056)
Supplement: S1 Table — Signal was distributed throughout sections, but predominantly identified in/around small-caliber blood vessels (lumen, endothelium, wall, or Virchow-Robbins space). Counts are averages from two reviewers. (DOCX) [file pone.0244056.s001.docx]

**S1 Table. Foci of probe hybridization in cases exhibiting ISH signal in sections of brain.** Signal was distributed throughout sections, but predominantly identified in/around small-caliber blood vessels (lumen, endothelium, wall, or Virchow-Robbins space). Counts are averages from two reviewers.

| Case | ISH Probe | Vessel-associated | Neuroparenchyma | Total |
| --- | --- | --- | --- | --- |
| 1 | UaCV | 279 | 193 | 472 |
|  | Neg. Control | 2.5 | 7.5 | 10 |
| 4 | UaCV | 119 | 45 | 164 |
|  | Neg. Control | 1 | 4.5 | 5.5 |
| 8 | UaCV | 74 | 19.5 | 93.5 |
|  | Neg. Control | 1 | 4.5 | 5.5 |
